# Clinical nomogram assisting in discrimination of juvenile dermatomyositis-associated interstitial lung disease

**DOI:** 10.1186/s12931-023-02599-9

**Published:** 2023-11-16

**Authors:** Minfei Hu, Chencong Shen, Fei Zheng, Yun Zhou, Liping Teng, Rongjun Zheng, Bin Hu, Chaoying Wang, Meiping Lu, Xuefeng Xu

**Affiliations:** Department of Rheumatology Immunology & Allergy Medicine, The Children’s Hospital, Zhejiang Univesity School of Medicine, National Clinical Research Center for Child Health, Hangzhou, 310003 PR China

**Keywords:** Interstitial lung Disease, Juvenile dermatomyositis, Diagnosis; nomogram

## Abstract

**Objective:**

To establish a prediction model using non-invasive clinical features for early discrimination of DM-ILD in clinical practice.

**Method:**

Clinical data of pediatric patients with JDM were retrospectively analyzed using machine learning techniques. The early discrimination model for JDM-ILD was established within a patient cohort diagnosed with JDM at a children’s hospital between June 2015 and October 2022.

**Results:**

A total of 93 children were included in the study, with the cohort divided into a discovery cohort (n = 58) and a validation cohort (n = 35). Univariate and multivariate analyses identified factors associated with JDM-ILD, including higher ESR (OR, 3.58; 95% CI 1.21–11.19, *P* = 0.023), higher IL-10 levels (OR, 1.19; 95% CI, 1.02–1.41, *P* = 0.038), positivity for MDA-5 antibodies (OR, 5.47; 95% CI, 1.11–33.43, *P* = 0.045). A nomogram was developed for risk prediction, demonstrating favorable discrimination in both the discovery cohort (AUC, 0.736; 95% CI, 0.582–0.868) and the validation cohort (AUC, 0.792; 95% CI, 0.585–0.930). Higher nomogram scores were significantly associated with an elevated risk of disease progression in both the discovery cohort (*P* = 0.045) and the validation cohort (*P* = 0.017).

**Conclusion:**

The nomogram based on the ESIM predictive model provides valuable guidance for the clinical evaluation and long-term prognosis prediction of JDM-ILD.

## Introduction

Juvenile dermatomyositis (JDM) is an uncommon immune-mediated systemic autoimmune vascular disorder characterized by symmetrical proximal muscle weakness, elevated serum muscle enzymes, and distinctive cutaneous manifestations such as Gottron papules and heliotrope rash [1]. This disease can also involve multiple internal organs, including the lungs, joints, heart, and gastrointestinal tract [2, 3]. In adults, interstitial lung disease (ILD) is a frequent complication of myositis, with a prevalence ranging from 30 to 50%, particularly prominent in Asian populations [4]. ILD in this context carries a substantial burden of morbidity and mortality, with rapidly progressive ILD being the leading cause of death [5, 6]. However, the prevalence of ILD in children with JDM is comparatively lower. The diagnostic evaluation primarily relies on imaging modalities such as chest computed tomography (CT), along with bronchoscopy and lung biopsy if necessary. And most children with JDM-ILD experience a chronic progressive course ILD [7, 8].

In children with JDM-ILD, initial lung involvement often presents as either asymptomatic or with mild symptoms. However, as the disease advances, clinical manifestations associated with ILD gradually emerge. Ultimately, there is a progressive decline in the functional units of the alveolar capillary system, which becomes challenging to reverse [8]. Therefore, early detection and timely intervention of JDM-ILD can significantly improve the long-term prognosis in children. Given the limited use of bronchoscopy and lung biopsy as invasive examinations in children, early diagnosis of JDM-ILD currently depends on chest high-resolution CT (HRCT) scans for assessing disease extent and monitoring progression [9–12]. However, the insidious nature of the onset of JDM-ILD makes early recognition difficult, and the radiation effects on children caused by multiple HRCTs over a certain period of time cannot be ignored. The pathogenesis of JDM-ILD is not clear, but numerous studies have suggested that its development involves multiple immune cells, cytokines and autoantibodies, and is highly associated with its own inflammatory activity [13, 14].

The primary goal of this study is to establish a clinical prediction model for JDM-ILD by analyzing relevant non-invasive clinical characteristics. The proposed model endeavors to assess the risk of JDM-ILD in affected patients, facilitate early detection of JDM-ILD, and predict the long-term prognosis by utilizing risk modeling. This approach enables personalized care for children with JDM-ILD, including tailored treatment plans and individualized follow-up strategies based on comprehensive risk model and prognostic assessment.

## Materials and methods

### Patients

Children with JDM hospitalized at the Children’s Hospital of Zhejiang University School of Medicine between June 2015 and October 2022 were retrieved. Patients with incomplete clinical information, including chest CT images, laboratory tests and follow-up data, were excluded from the study. Additionally, the JDM patients were aged under 16 years and were followed for at least 6 months. The study was approved by the Ethic Review Board of Children’s Hospital, Zhejiang University School of Medicine (No.2022-IRB-082). In accordance with the Helsinki declaration, informed consent was waived as the data were anonymized and de-identified prior to analysis, and the study was determined to pose no additional risk to patients.

### Diagnostic criteria

The diagnosis of JDM was made according to the 2017 European League Against Rheumatism/American College of Rheumatology (EULAR/ACR) classification criteria [15]. The children with ILD were diagnosed according to the presence of the specific indicators, including respiratory symptoms (e.g., cough, shortness of breath), respiratory signs (e.g., pulmonary rales, pestle fingers), hypoxemia, and abnormal chest HRCT imaging (e.g., consolidations, reticulations, honeycombs) [16]. HRCT scans of the patients were independently evaluated by two expert radiologists in the study. Additionally, a meticulous evaluation process involves excluding congenital, metabolic, infectious, and neoplastic factors that may potentially contribute to the development of ILD.

### Discovery and Validation cohorts

**Fig. 1:**
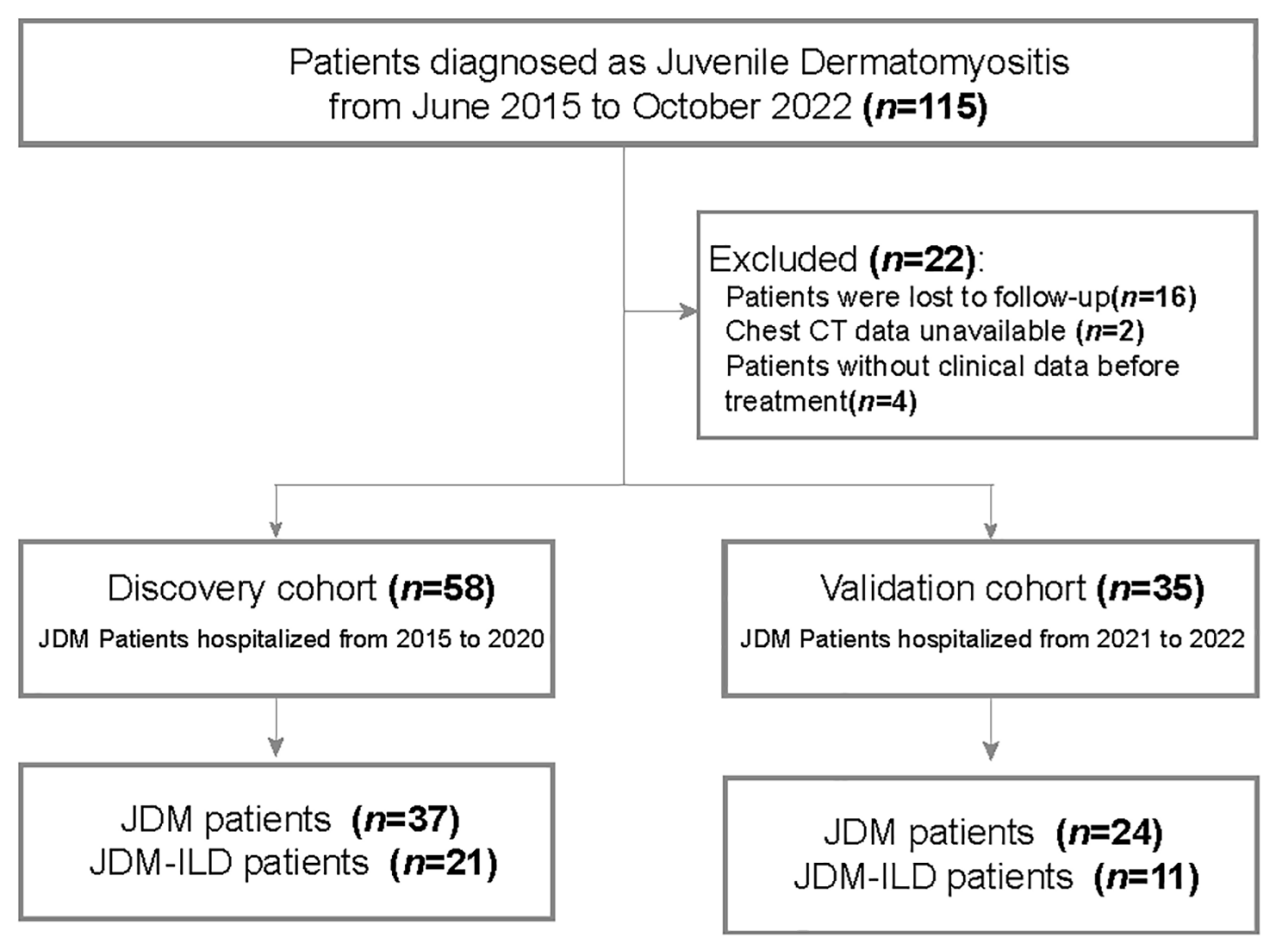
Study Design Flow Diagram. Flowchart illustrating the research recruitment and categorization

The clinical and laboratory data for the patients were extracted through the retrieval of their medical records. Children with JDM prior to 2021 were categorized as the discovery cohort, while those diagnosed with JDM between 2021 and 2022 were designated as the validation cohort (Fig. 1). At the follow-up after treatment, children who exhibited moderate improvement in dimensions physician global activity, patient global activity, manual muscle testing, health assessment questionnaire, enzyme and extramuscular activity, along with improved lung abnormalities as indicated by HRCT, were classified as having a favorable prognosis [17]. Otherwise, other children were categorized as the poor prognosis, especially worsening during the follow-up, including physician-assessed or extramuscular organ disease activity worsening by 2 cm on a 10-cm VAS, muscle testing worsening 20%, any 3 of 6 IMACS core set activity measures worsening by 30%, or evidence of deteriorating lung lesions on HRCT of the chest [18]. The primary outcome was worsening risk within one year.

### Statistical analysis

**Fig. 2:**
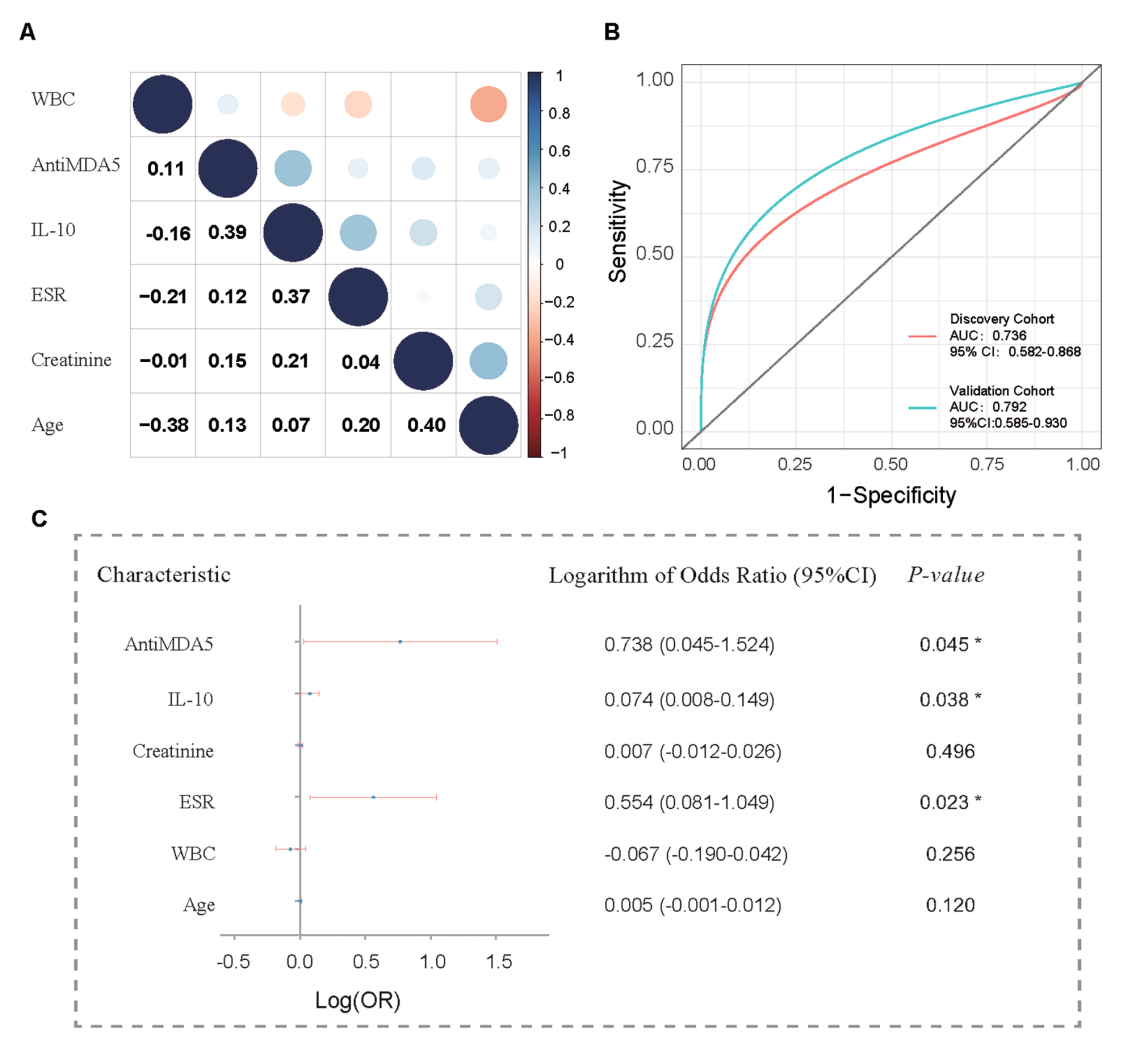
Analysis of clinical characteristics and discrimination of the predictive model. **A** shows the correlation of the extracted clinical features. **B** displays the receiver operating characteristic (ROC) curves of the predictive model in the discovery and validation cohorts. **C** presents a forest plot showing odds ratio (OR) calculated by logistic regression analysis for the independent risk factors

At first, logistic regression was used to extract clinical features significantly associated with the occurrence of ILD in patients with JDM. Spearman’s correlation analysis was produced to evaluate the extracted features. If spearman’s correlation coefficient ≥ 0.80, the features was considered redundant and excluded (Fig. 2A). Secondly, the unrelated clinical features identified were utilized to established a clinical prediction nomogram. The performance of the predictive nomogram was evaluated in terms of discrimination, calibration, and clinical utility. Discriminative ability was assessed using receiver operating characteristic (ROC) curves, with the area under the curve (AUC) as the measure. To obtain robust estimates, bootstrapping with 2000 replicates was performed to generate AUCs and their corresponding 95% confidence intervals (CIs). Decision curve analysis (DCA) was developed to assess clinical usefulness. Finally, patients in the discovery cohort were stratified into low-score and high-score subgroups based on the median nomogram score. Analysis was conducted using the Kaplan-Meier method to estimate time-to-event data.

Normally distributed continuous variables were examined as mean ± standard deviation (SD) and compared using t-tests. Other non-normally distributed continuous variables were described as the interquartile range (IQR) and analyzed using the Mann-Whitney U test. Categorical variables were expressed as percentages and compared using the chi-square test or Fisher’s exact test. In R 4.0.5 software(http://www.R-project.org), Kaplan-Meier curves were created using the survival package, statistical analyses were conducted using the STATs package, and the clinical discriminative model’s nomogram was implemented using the RMS package. A two-side *P*-value < 0.05 was deemed statistically significant.

## Results

### Characteristics of the included children with JDM

A total of 93 children were included in this study, with the cohort divided into a discovery group (n = 58 [62.37%], diagnosed between 2015 and 2020) and a validation group (n = 35 [37.63%], diagnosed between 2021 and 2022). The median age (IQR) of all patients was 84 months (58–120 months), and there were 48 boys (51.61%) among them. There was no significant difference in basic clinical data between discovery and validation cohorts. The study observed various chest HRCT imaging features in children with JDM-ILD, including ground glass-like changes (n = 15), reticular and linear changes (n = 13), lobular septal thickening (n = 9), pleural thickening (n = 10), nodular cloudiness (n = 7), bronchial wall thickening (n = 7), and cystic changes (n = 3).

In terms of treatment, conventional dose corticosteroids were administered to all 93 (100%) children as the primary therapeutic intervention. Additionally, high dose corticosteroids were employed in 23 (24.73%) children, while 59 (63.44%) children received immunoglobulin therapy. Most patients had received at least one line of immunosuppressive drugs in combination with steroids, including methotrexate (n = 61,65.59%), hydroxychloroquine (n = 17,18.28%), cyclophosphamide (n = 5,5.38%), baricitinib (n = 4,4.30%). Notably, all included children with available follow-up data in this study experienced favorable outcomes, as there were no reported deaths. During the follow-up period, a total of 18.00 children (31.03%) in the discovery cohort and 12.00 children (34.29%) in the validation cohort were classified as having a poor prognosis. Meanwhile, other descriptive characteristics of all the patients are summarized in Table 1.

**Table 1:** Characteristics of the patients with JDM^1^ before treatment

	All patients *n* = 93, No. (%)	Discovery cohort *n* = 58, No. (%)	Validation cohort *n* = 35, No. (%)	*P* -Value
Age, Median (IQR), month	84.00(58.00-120.00)	46.50(24.50-60.75)	69.00(23.00-83.50)	0.0488*
Height, Mean (SD), cm	122.16(21.23)	120.58(22.89)	124.77(18.14)	0.359
Weight, Mean (SD), kg	26.01(11.98)	25.77(12.48)	26.40(11.27)	0.807
BMI, Mean (SD), kg/m2	16.68(2.95)	16.94(2.89)	16.24(3.04)	0.274
Sex				
Male	48(51.61)	27.00(46.55)	21.00(60.00)	0.297
Female	45(48.39)	31.00(53.45)	14.00(40.00)	
CMAS, Mean (SD), score	37.96(8.02)	38.52(7.13)	37.03(9.34)	0.389
WBC, Mean (SD), /µL	7.48(2.84)	7.52(3.32)	7.41(1.83)	0.843
Neutrophil, Median (IQR), /µL	3.86(2.66–5.49)	3.91(2.37–5.70)	3.84(3.13–5.18)	0.883
Hemoglobin, Mean (SD), g/L	119.86(13.97)	119.28(13.19)	120.83(15.34)	0.606
Blood platelet, Mean (SD),×109 /L	283.20(82.72)	277.47(82.15)	292.71(83.98)	0.392
ALT, Median (IQR), U/L	50.00(24.00–98.00)	50.00(25.50–97.50)	41.00(21.50–95.00)	0.827
Creatinine, Mean (SD), µmol/L	36.58(13.85)	42.79(12.91)	26.29(8.06)	< 0.01**
CRP, Median (IQR), mg/L	0.65(0.50–2.82)	1.10(0.50-3.00)	0.50(0.49–1.04)	< 0.01**
ESR, Median (IQR), mm/h	15.00(9.00–25.00)	16.00(9.00-25.75)	15.00(10.50–23.00)	0.799
In reference range	65(69.90)	40(68.97)	25(71.43)	0.802
Outside reference range	28(30.10)	18(31.03)	10(28.57)	
CK, Median (IQR), U/L	306.00(92.00-1002.00)	256.00(83.50-1628.75)	336.00(155.00-714.00)	0.806
CK-MB, Median (IQR), U/L	30.00(23.00–69.00)	32.50(25.00-77.25)	28.00(21.00–69.00)	0.301
IL-2, Mean (SD), pg/ml	2.57(1.10)	2.61(1.29)	2.51(0.70)	0.609
IL-4, Median (IQR), pg/ml	2.30(1.80-3.00)	2.30(1.80–3.40)	2.20(1.90–2.65)	0.369
IL-6, Median (IQR), pg/ml	8.40(3.50–14.30)	6.05(2.32–11.57)	9.40(6.25–21.10)	< 0.01**
IL-10, Median (IQR), pg/ml	5.30(3.70–7.40)	5.40(3.62–7.07)	4.90(3.70-8.00)	0.763
TNF-α, Median (IQR), pg/ml	1.60(1.20–2.30)	1.60(1.20–2.30)	1.40(1.20–2.10)	0.526
INF-γ, Median (IQR), pg/ml	2.70(1.70-4.00)	3.20(1.92–5.72)	2.00(1.50–2.95)	< 0.01**
Fever	31(33.33)	22.00(37.93)	9.00(25.71)	0.325
Erythra	87(93.55)	54.00(93.10)	33.00(94.29)	0.822
ANA positive	38(40.86)	25.00(43.10)	13.00(37.14)	0.727
Myositis antibody positive				
Anti-TIF1	5(5.38)	3.00(5.17)	2.00(5.71)	0.911
Anti-NXP2	9(9.68)	3.00(5.17)	6.00(17.14)	0.126
Anti-MDA5	15(16.13)	9.00(15.52)	6.00(17.14)	0.836
Anti-Jo1	2(2.15)	0.00(0.00)	2.00(5.71)	0.270
Anti-Ro52	14(15.05)	7.00(12.07)	7.00(20.00)	0.461
Anti-U1RNP	4(4.30)	4.00(6.90)	0.00(0.00)	0.289
ILD	32(34.41)	21.00(36.21)	11.00(31.43)	0.807

Abbreviations: JDM, juvenile dermatomyositis; BMI, Body Mass Index; CMAS, childhood myositis assessment scale; WBC, white blood cell; ALT, alanine aminotransferase; ESR, erythrocyte sedimentation rate; CRP, C-reactive protein; CK, creatine kinase; CK-MB, creatine kinase isoenzymes; ANA, antinuclear antibodies; ILD, interstitial lung disease;

SI conversions: WBC to×10^9^ per liter, multiply by 0.001; CK to microkatals per liter, multiply by 0.0167

^1^ All JDM patients meet the 2017 EULAR/ACR classification criteria

### Predictors of JDM-ILD

Among the investigated patients in the study, univariate logistic regression analysis of clinical characteristics revealed that several factors were sequentially associated with an increased likelihood of developing JDM-ILD. These factors included older age of diagnosis (OR, 1.02; 95% CI 1.01 to 1.03), lower white blood cell counts (OR, 0.83; 95% CI 0.69 to 0.99), higher ESR (≥ 20 mm/h, OR, 4.63; 95% CI 1.84 to 12.14), higher creatinine levels (OR, 1.03; 95% CI 1.00 to 1.07), higher IL-10 levels (OR, 1.24; 95% CI 1.11 to 1.43), and positivity for MDA-5 antibodies (OR, 7.46; 95% CI 2.28 to 29.36). In the multivariate regression analysis, higher ESR (≥ 20 mm/h, OR, 3.58; 95% CI 1.21 to 11.19, *P* = 0.023), higher IL-10 levels (OR, 1.19; 95% CI, 1.02–1.41, *p* = 0.038), positivity for MDA-5 antibodies (OR, 5.47; 95% CI, 1.11–33.43, *P* = 0.045) were found to be significantly associated with JDM-ILD (Fig. 2C). Furthermore, the linear regression model using ESR, IL-10, and MDA-5 antibody as predictors showed a significant discriminatory power with an AUC of 0.736(95% CI,0.582–0.868) in discovery cohort and 0.792(95% CI,0.585–0.930) in validation cohort (Fig. 2B).

### Nomogram and model assessment

**Fig. 3:**
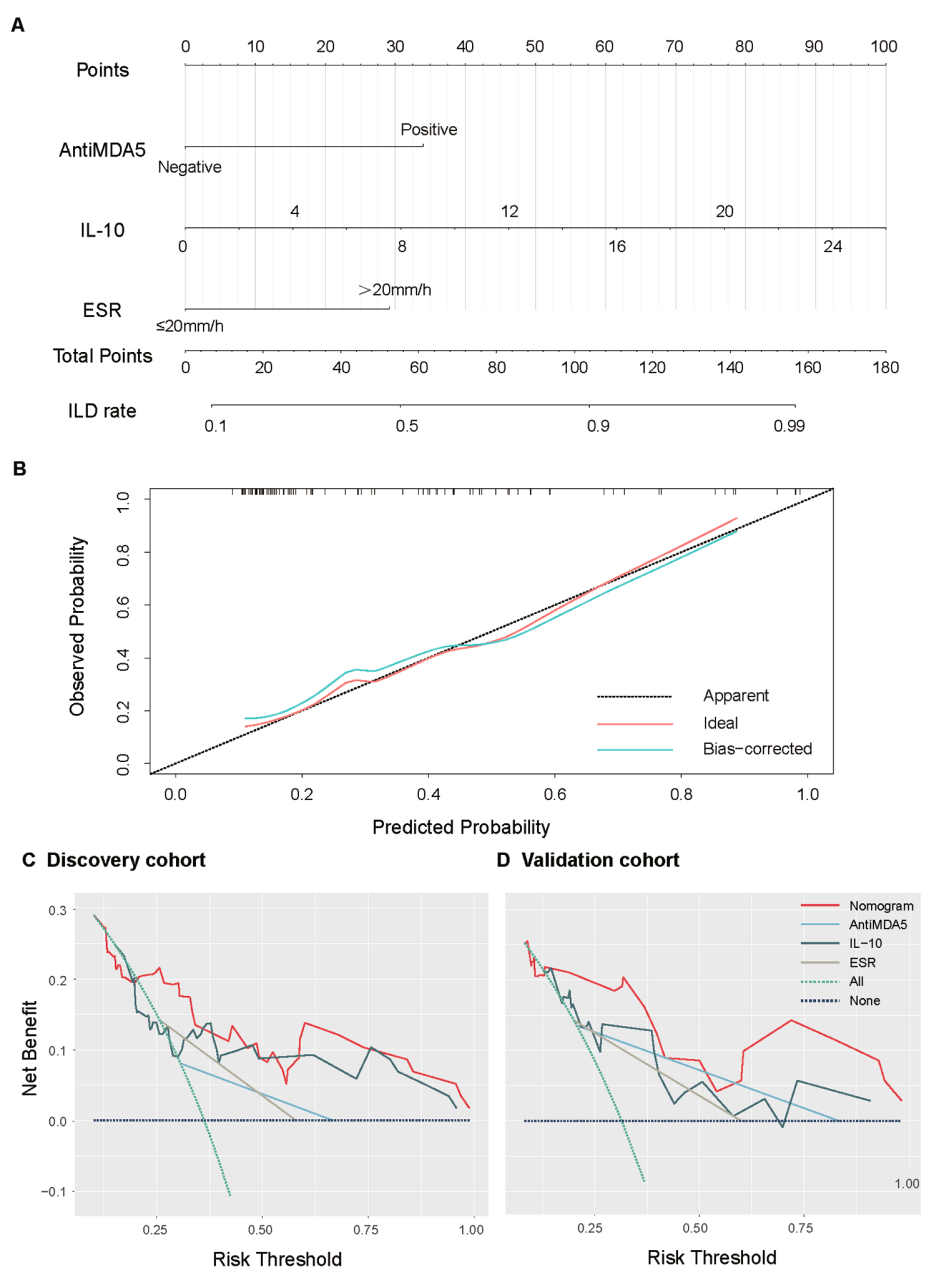
Construction of clinical discriminative nomogram and decision curves analysis (DCA). **A** displays the nomogram combining associated clinical factors to estimate the risk of developing JDM-ILD. **B** shows the bootstrapped estimates of calibration accuracy for the nomogram. Assessing the clinical usefulness of the prediction model and other indicators in the discovery cohort (**C**) and validation cohort (**D**)

A nomogram was developed based on the linear regression model mentioned above to predict the probability of JDM-ILD (Fig. 3A). Higher total points derived from the sum of the assigned number of points for each predictor were associated with the risk of JDM-ILD. Meanwhile, the calibration plot exhibited a favorable agreement in predicting JDM-ILD via bootstrap resampling (Fig. 3B). The DCA revealed that the prediction model provided greater net benefits compared to both the treat-all-patients and treat-no-patients schemes across all threshold probabilities. Moreover, the prediction model demonstrated superior net benefits compared to other indicators when assessing the clinical utility in the discovery cohort (Fig. 3C) and the validation cohort (Fig. 3D).

### Nomogram score and Disease progression

**Fig. 4:**
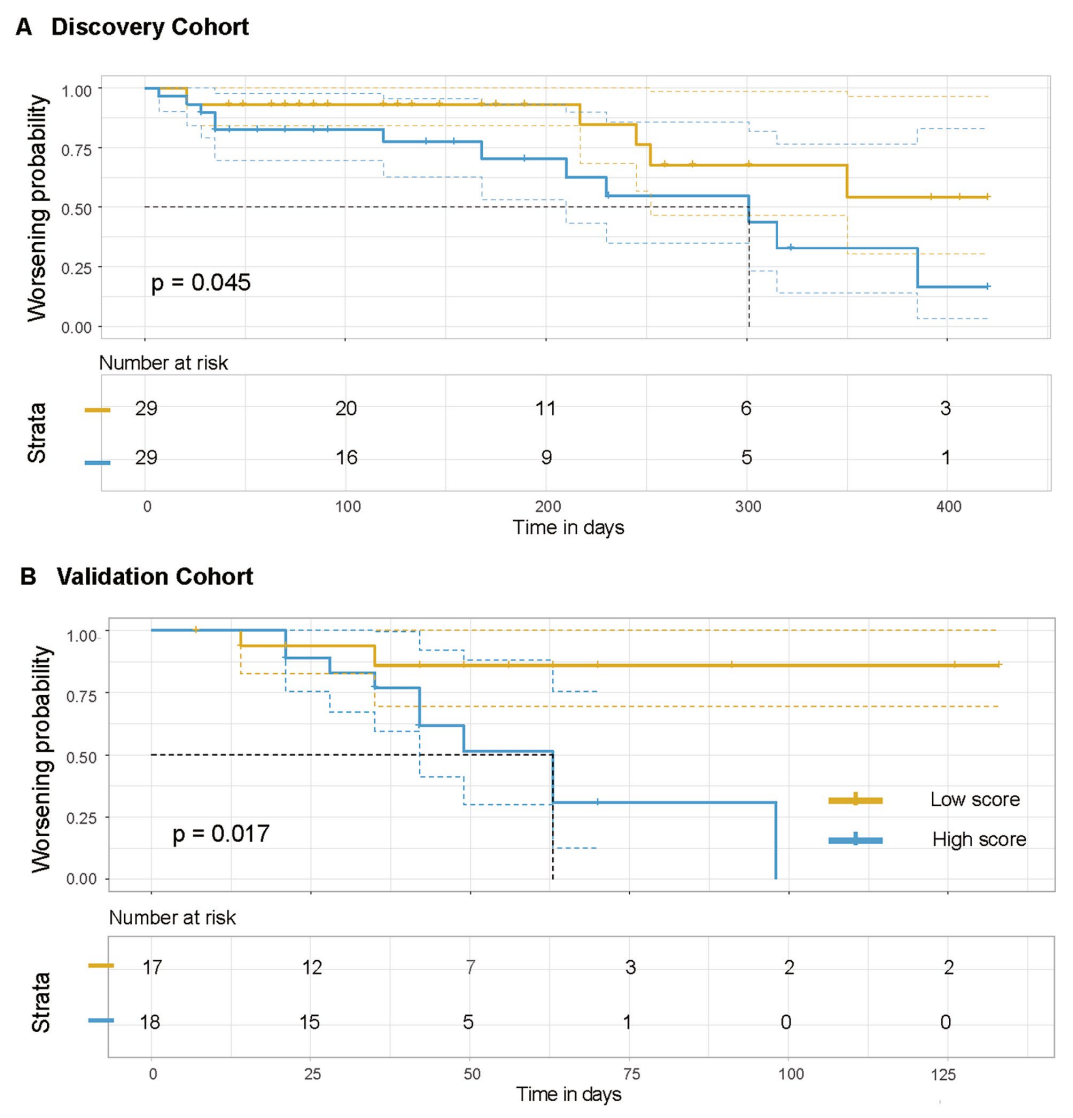
Survival analyses based on different nomogram scores. Patients in the low nomogram score group demonstrated significantly improved probabilities of disease deterioration in the discovery cohort (**A**) and validation cohort (**B**)

Based on the median nomogram score (score = 0.224) in the discovery cohort, the enrolled patients were categorized into two groups: the low score group (n = 29 in the discovery cohort, n = 17 in the validation cohort) and the high score group (n = 29 in the discovery cohort, n = 18 in the validation cohort). Patients in the high score group were found to be associated with a higher risk of disease progression (Cochran-Armitage test for trend: *p* = 0.0024). The survival analyses demonstrated a statistically significant association between the high score group and elevated probabilities of disease deterioration, observed in both the discovery cohort (*P* = 0.045, Fig. 4A) and the validation cohort (*P* = 0.017, Fig. 4B).

## Discussion

ILD is a prevalent and severe complication in children with JDM, significantly impacting their quality of life and prognosis, particularly in anti-MDA5 positive JDM patients [19–21]. While the incidence of ILD in JDM is relatively lower compared to adult DM, [22] the development of ILD in children can result in profound and irreversible pulmonary impairments [20]. In this retrospective study, three clinical features, including ESR, IL-10, and MDA-5 antibody, were extracted to construct a discriminative nomogram. The model exhibited strong predictive performance in assessing the risk of JDM-ILD. Additionally, the model demonstrated notable clinical utility and provided prognostic information for JDM in clinical practice. We propose ESIM, a predictive model utilizing the fitted discriminative nomogram of ESR, IL-10, and MDA-5 antibody, for assessing the risk of developing JDM-ILD. The implementation of the ESIM model has the potential to facilitate early detection and individualized treatment approaches for children with JDM-ILD.

Myositis-specific antibodies, including NXP2, MDA5, Jo1, etc., have gained prominence in the clinical distinction of dermatomyositis. MDA5, encoded by the IFIH1 gene, [23] is reported to be positive in approximately 11–60% of dermatomyositis cases, with a positivity rate of 6-12% in children. Notably, MDA5 antibody positivity is more prevalent in Asian populations compared to white populations [24]. The anti-MDA5 antibody serves as a valuable biomarker for ILD in JDM and can also predict ILD complications [25]. Consistent with previous studies, the study also revealed a significant association between MDA5 positivity and the presence of JDM-ILD in children. Patients with MDA5-positive DM are at a high risk of developing rapidly progressive interstitial lung disease (RP-ILD) and have a poor prognosis, with an early-stage mortality rate of approximately 50% [23, 26, 27]. In our study, the occurrence of RP-ILD leading to mortality was rare. These findings also suggest that children with MDA5-positive JDM-ILD may have a more favorable prognosis compared to adults with DM.

JDM is a chronic systemic autoimmune disease associated with the involvement of various inflammatory factors. In DM-ILD patients, particularly those positive for MDA-5 antibodies, researchers have observed elevated levels of interleukin, specifically IL-6 and IL-10, which are pro-inflammatory cytokines [28, 29]. These cytokines are closely linked to disease activity and have the potential to induce alveolar epithelial cell injury through macrophage activation or other pathways, leading to the development of pulmonary fibrosis and subsequent ILD [30]. ESR serves as an unspecific biomarker of the acute phase response, offering valuable information during the active phase of JDM [31, 32]. ESR has been proposed as a serum indicator for assessing disease activity and facilitating early discrimination in JDM [33, 34]. However, some researchers have been suggested that the elevated ESR in DM patients is not directly correlated with the degree of inflammatory muscle damage but may instead indicate the severity of pulmonary involvement [35]. The secretion of cytokines by inflammatory cells in the muscle tissue of children with JDM is minimal, and detectable levels of cytokines and ESR are observed only when ILD is present [36]. The study demonstrated a significant elevation of IL-10 and ESR in JDM-ILD patients. The early discriminant model incorporated IL-10 and ESR as important factors, particularly in children with positive MDA-5 antibodies. We have incorporated three independent risk factors into a novel discriminative model, the ESIM model, for the purpose of risk assessment in JDM-ILD. Based on the assessment of the ESIM model, we evaluated a cut-off value of 88 points for clinical application in diagnosing JDM-ILD. For instance, if a JDM patient has a positive anti-MDA5 antibody (34 points), an ESR ≥ 20 mm/h (29 points), and an IL-10 ≥ 6.9 pg/ml (25 points), their total score would reach 88 points, indicating the requirement for meticulous clinical surveillance of concurrent ILD. Currently, there is no consensus on the standardized screening of ILD in children with JDM. Pulmonary function tests and chest HRCT are useful tools, but their interpretation and timing of review are still controversial [37]. Thus, identifying high-risk groups based on the ESIM model at the time of diagnosis is essential.

Despite the low mortality rate in children with JDM-ILD, it is essential to prioritize the long-term lung effects and quality of life of these patients. Currently, there is a lack of well-defined and individualized treatment strategies for children with JDM-ILD. Our discriminant model offers valuable insights into disease severity, aiding clinical decision-making and personalized treatment strategies. The nomogram score derived from the model serves as a prognostic indicator, enabling the development of individualized follow-up plans. Higher scores indicate the need for more frequent monitoring to promptly identify and address potential complications.

This study has several limitations. Firstly, although previous studies have identified additional risk factors such as positive anti-Jo-1 antibody and elevated CRP for the development of complicated ILD, [38] our study did not find significant differences in these indicators. Given the limited number of studies focused on children compared to adults, it is essential to expand the sample size in future investigations. Secondly, assessing the prognosis of children with JDM-ILD only based on mortality is challenging, as the incidence of JDM-ILD progressing to rapidly progressive ILD leading to death is much lower in children. Furthermore, the subjective nature of assessing deterioration in children used in our study may introduce bias into the results. Thirdly, our sample was drawn from a single treatment center, which may limit the generalizability of the findings to the broader population. Lastly, the relatively rare onset of JDM-ILD resulted in some children not being screened for MSA, and their data were considered negative by default, potentially introducing bias into the analysis. Future research should address these limitations to enhance the robustness and generalizability of the findings.

### In conclusion

This study established a discriminative nomogram for JDM-ILD based on the ESIM model including ESR, MDA-5, and IL-10 in enrolled children, providing clinical guidance for evaluating JDM-ILD and predicting long-term prognosis.
